# An evaluation of the social deprivation practice grant in Irish general practice

**DOI:** 10.3399/BJGPO.2023.0195

**Published:** 2024-06-26

**Authors:** Muireann O Shea, Bridget Kiely, Patrick O’Donnell, Susan M Smith

**Affiliations:** 1 Discipline of Public Health and Primary Care, Trinity College, Dublin, Ireland; 2 Department of General Practice, Royal College of Surgeons in Ireland University of Medicine and Health Science, Dublin, Ireland; 3 School of Medicine, University of Limerick, Limerick, Ireland

**Keywords:** family medicine, general practice, inequalities, health inequities, Ireland

## Abstract

**Background:**

The inverse care law states that availability of good medical care varies inversely with the need for it in the population served. In 2019, the main medical union and the Department of Health in the Republic of Ireland (RoI), agreed on funding a social deprivation practice grant for general practices in urban deprived areas.

**Aim:**

To examine the implementation and impact of the social deprivation practice grant in participating general practices.

**Design & setting:**

A mixed-methods study with sequential design based in Irish general practice.

**Method:**

Data were collected using a questionnaire and online semi-structured interviews with GPs and practice staff. Data were analysed separately, and the findings compared to examine the extent to which they converged or diverged.

**Results:**

There were 25 survey responses and nine interviews. All practices reported the grant was beneficial and most practices utilised the grant to fund additional doctor hours (17/25). Both surveys and interviews indicated that a small amount of additional funding allowed additional clinical need in areas of deprivation to be addressed, but there were some barriers identified in accessing the grant and implementing planned expenditure.

**Conclusion:**

Delivery of health care in areas of socioeconomic deprivation presents significant challenges. While there were some problems with implementation, the introduction of a small, targeted grant for general practices in areas of social deprivation allowed those practices to enhance their services, with tailored initiatives seeking to meet the needs of their patient populations.

## How this fits in

The inverse care law states that availability of good medical care varies inversely with the need for it in the population served. In the Republic of Ireland (RoI), allocation of state-funded GP services is based on the size of a population rather than the level of need – therefore, general practices in areas of deprivation receive similar funding to those in more affluent areas per patient. While very modest in terms of resource allocation, the social deprivation grant represents the first acknowledgement in the Irish health service of additional needs in these areas. This is the first study of a funding initiative specifically allocated to support GP services for communities with a high degree of social deprivation in the RoI.

## Introduction

Julian Tudor Hart’s inverse care law stated that ‘*the availability of good medical care tends to vary inversely with the need for it in the population served*’,^1^ and this is borne out in aspects of the Irish health service. It is well established that multimorbidity and polypharmacy are more prevalent in areas of social deprivation.^2^ The onset of multimorbidity occurs 10–15 years earlier in patients from areas of highest deprivation compared with the least deprived areas.^3^ Mental health issues are more prevalent in people with increasing numbers of physical disorders and this association has a consistent social gradient.^4^ Patients living in areas of socioeconomic deprivation have higher care needs and healthcare utilisation.^5,6^ A survey of Irish GPs in 2005 found that 22%–30% of GPs were working in areas of deprivation.^7^ An analysis of the relationship between multimorbidity, GP resources, and workload by deprivation in Scotland found that GP consultation rates showed a clear association with deprivation.^8^

The majority of GPs in the Republic of Ireland (RoI) run mixed public–private practices. Private patients pay to attend a general practice, public patients are funded by the government as part of the General Medical Services (GMS) contract. The current allocation of medical funding in the GMS scheme is based on a capitation model. Funds are distributed according to the number of GMS patients registered to a practice, with variable rates based on patient age and no recognition of differential levels of need of the patients attending the practice. This means that GP practices in areas of deprivation receive similar funding per patient as those in more affluent areas. A UK review identified that practices serving more socioeconomically deprived areas received 7% less funding per need-adjusted registered patient than those serving less deprived patient populations, despite the existence of weighted capitation payments taking account of socioeconomic deprivation.^9^

In 2019 an agreement was reached between the Irish Medical Organisation (IMO), Health Service Executive (HSE), and Department of Health to allocate funding to support GP services for communities with a high degree of social deprivation. The IMO is the main trade union for negotiation of contractual arrangements for GPs in the RoI. The HSE is responsible for the delivery of public health services in the RoI; the Department of Health develops relevant policies. Under this agreement, each approved practice could receive an allowance of between €7500 and €12 500 per annum (approximately between 6450.50 GBP and 10 752.50 GBP). Minimum application criteria to qualify for the grant included the practice should be in an urban area, have a minimum of 350 GMS patients, and 200 patients living in disadvantaged areas. The process was open to all potentially eligible GMS contract-holding general practices that completed an application, which was based on a time-consuming process involving mapping of all their patient addresses based on Pobal Index maps^10^ and reporting the proportion of their GMS patients living in disadvantaged, very disadvantaged, and extremely disadvantaged areas. The grant could be utilised to provide additional services to their patients.^11^

The aim of this study was to examine the initial implementation and impact of the social deprivation practice grant in Irish general practice.

## Method

A sequential mixed-methods research approach was employed. Qualitative and quantitative data were collected through a GP questionnaire, and the questionnaire responses were used to inform the design of the interview questions. The data were analysed separately, and the findings compared to examine the extent to which results converged or diverged.

Questionnaire data collected included practice demographics, resources funded by the grant, benefits to practice and patient, and the grant application process. There was also an opportunity to provide free-text comments in the online questionnaire. Semi-structured interviews were used to gain a deeper understanding of GPs’ experiences in applying for and utilising the social deprivation grant.

### Sampling

For the quantitative data collection, there was no publicly available record of the practices in receipt of the grant so all potentially eligible practices in receipt of the social deprivation grant were invited to participate by email through gatekeeper organisations including Deep End Ireland (coordinator is co-author Professor Susan Smith) and the IMO. A study information leaflet, consent form, and practice questionnaire were developed and emailed to all potentially eligible practices through the gatekeepers in compliance with General Data Protection Regulation (GDPR). A reminder email was sent 2 weeks after the initial correspondence.

On completion of the online survey, practices were invited to submit a contact email address if they were willing to participate in a semi-structured interview. We adopted a pragmatic approach to the sample size for the qualitative element of the study, which was based on the need to collect sufficient information to address the study aims and include an appropriate range of contributions. Information power was determined by the study aims, sample specificity, quality of the interview dialogue, and case analysis.

### Interviews

A convenience sampling approach was utilised for qualitative data collection with study participants, who completed the questionnaire opting in to complete a follow-up interview. Interview questions were designed within the main research objectives and the qualitative questionnaire data were utilised to inform the design of the interview schedule. Each participant interview was recorded, and detailed notes taken to ensure completeness and accuracy. All interviews were conducted and transcribed by MOS, a female GP and lead study author.

### Data analysis

Descriptive statistics were used to summarise and present key findings. Analysis of variance and *t*-tests, as appropriate, were used to evaluate if there were significant differences between means across various groups.

Qualitative data, including free-text comments and interview data, were analysed using thematic analysis supported by NVivo software (version 12). Major themes were supported by sub-themes to enable more granular data descriptions and explanations to be presented. Data were charted according to the data analytic framework and a narrative was produced for each theme and sub-theme.^12,13^ Qualitative and quantitative data were integrated using a triangulation protocol^14^ to assess for convergence, complementarity, and dissonance. Identified themes were coded as agreement, partial agreement, silent, or dissonant.

## Results

### Survey results

The total number of practices in receipt of the grant was not made publicly available but given the funding amount and size of grants awarded, it is likely that there were 100–150 practices in total. There were 25 survey responses. The mean number of patients per practice was 5147. The mean number of patients per full-time equivalent GP was 1967. An average of 22% of patients lived in an area of disadvantage per practice. One hundred per cent of practices responded that the grant had been of benefit. Analysis of variance and *t*-tests did not identify significant differences between means across various groups.

Twenty per cent found the application process easy compared with 36% finding it difficult or very difficult.

Many practices used the grant to fund additional doctor (17/25) and nursing (6/25) hours. Other resources funded by the grant included counselling, addiction counselling, link worker, psychology, interpreter services, paying for the first month of ExWell Medical (structured exercise programme) classes for patients, reception and administration staff, nursing home cover, extended mental health consultations, health promotion and screening, and a medical clinic for complex patients.

### Qualitative results

Seven GPs and two practice managers volunteered to participate in the interviews. Analysis of the qualitative data identified 13 sub-themes, which were grouped into four main themes. The main themes identified were as follows: benefits for practice and patient; complexities of care; challenges to providing care in areas of deprivation; and barriers to applying for and implementing the grant.

#### Benefits for practice and patient

Participants reported benefits to the practice including reduced workload because of the resources funded by the grant, better access to and quality of GP care, and reduced risk of burnout among staff.

*‘From the GP’s perspectives there was better continuity of care, and they were able to focus on more than one issue in a longer consultation, so they felt they were giving better care to patients*.’ (Interview 3)

*‘It reduced the burden on the doctors working in the practice who were experiencing some symptoms of burnout due to the constant challenge of being the only open and accessible resource to the patients during COVID challenges.’* (Questionnaire 13)

#### Complexities of care

A recurrent theme in the data was that of the complex nature of patient presentations within areas of deprivation and the prevalence of comorbidities and mental health issues.

*‘All patient interactions now longer, more complex, with a huge burden of psychological illness.’* (Questionnaire 22)

' *… there was so much pressure on GPs with consults taking longer due to social issues and mental health issues.*’ (Questionnaire 5)

Many participants discussed the need for longer consultation times to improve quality of care and avoid ‘*multiple fragmented consultations’* (Interview 5). Participants identified specific areas of patient care that had been improved because of the grant including access to doctor appointments, avoidance of crisis presentations, enhanced focus on screening, and chronic disease management.

*‘It did improve access to appointments because it freed up the partners to see complex patients.*’ (Interview 4)

*‘I think there has probably been a lot less crisis presentations than there would have been without it*.’ (Interview 1)

*‘It has given us the opportunity to be proactive rather than reactive in the health management of a vulnerable population. We are aware that there is lower uptake of screening and preventive medicine in this cohort. We aim to improve this.’* (Questionnaire 7)

#### Challenges to providing care in areas of deprivation

Participants identified challenges to providing care in areas of deprivation including health inequities and a high prevalence of mental health issues and multimorbidity. Interviewees described the additional time and resources required to manage a combination of multimorbidity, mental health, and social issues.

'*The demands on our time and resources are far greater than in other areas as our patients have diseases associated with deprivation and diseases made harder to treat by conditions associated with deprivation such as smoking, addiction, homelessness, social/family crisis, lack of education or understanding about their conditions.*’ (Questionnaire 7)

#### Barriers to applying for and implementing the grant

Participants highlighted difficulties relating to the grant application process, assessment tools, payment uncertainty and/or delays, and recruitment. Some practices felt the Pobal Index^10^ (national deprivation index) did not accurately represent their patient population.

*‘It was clear that the Pobal Deprivation maps exclude a lot of our severely deprived patients.*’ (Interview 18)

'*Implementing expenditure has been very challenging due to the uncertainty around the amount of funding available, the guaranteed continuation of said funding and the gaps in funding allocation.’* (Questionnaire 25)

The application process was cited as being time-consuming and a potential barrier to application.

*‘I think an awful lot of practices that were really deprived didn’t apply because it’s too much hassle and they didn’t know about it.’* (Interview 5)

A recurring theme was that of difficulty in recruiting and retaining staff.

*‘Trying to retain really good staff is very challenging with the way the grant was being implemented*.’ (Interview 6)

'*Nobody wants to work in areas of deprivation.*’ (Interview 7)

Integration of both qualitative and quantitative data identified agreement that additional resourcing of practices in areas of deprivation improved healthcare delivery, that more doctor and nurse hours are required to address complex patient needs in areas of deprivation, and that the application process could be simplified.

## Discussion

### Summary

Delivery of health care in areas of socioeconomic deprivation presents significant challenges. GPs working in these areas manage higher levels of multimorbidity, mental health and social issues, complex care needs, and increased healthcare utilisation.^1,3,6^

The study data indicated that the additional resources from the social deprivation grant were beneficial to both patients and practice staff working in areas of deprivation. These monies allowed practices to enhance the service they provide to their patients while reducing workload for the current practice staff. However, participants identified a need for ongoing funding and a simplified application process.

### Strengths and limitations

This is the first study of a funding initiative specifically allocated to support GP services for communities with a high degree of social deprivation in the RoI. Triangulation of the quantitative and qualitative data provided the opportunity to gain a deeper understanding of the GP team’s experience of the initiative.

All eligible practices in receipt of the social deprivation grant in Irish general practice were invited to participate through gatekeeper organisations but we were unable to ascertain how many practices in total are in receipt of the grant. The relatively small sample size and low response rate is a limitation; however, the qualitative data adds depth to the collected survey data.

Participants volunteered to take part and recruitment was through the IMO and Deep End Ireland – therefore, it is possible those who volunteered to participate are more actively involved in patient advocacy resulting in selection bias. A further limitation was the lack of analysis of the impact of the grant-funded activity on patient outcomes, which was beyond the scope of the current study.

### Comparison with existing literature

The social deprivation grant was the first acknowledgement in the Irish health service of the need for additional resources for general practice in areas of socioeconomic deprivation. Evidence from literature in the UK identifies a higher burden of disease and healthcare utilisation patterns in areas of socioeconomic deprivation.^3,4^ Funding structures have been introduced to address these issues and include the Quality and Outcomes Framework (QOF) and weighted capitation payments introduced to the UK GMS contract in 2004. Evidence showed an initial narrowing of the gap in care provision between least and most deprived practices using QOF indicators;^15,16^ however, further studies suggest that this initial improvement has not sufficiently addressed health inequalities.^16–18^ Recent analysis by The Health Foundation in the UK concluded that, despite attempts to address deprivation based on weighting of capitation payments, underfunding relative to need may be a widespread problem across all major funding flows into general practice in the NHS and called for additional funding of general practice in areas of deprivation to help address health inequalities.^9^

The introduction of a new Scottish GMS contract in 2018 saw the use of a funding formula, which allowed for greater weighting of practice payments for deprivation, formation of ‘GP clusters’, and expansion of the multidisciplinary team.^19^ To the author’s best knowledge, a formal quantitative evaluation of the impact of these changes has not yet taken place; one qualitative study^20^ reports limited progress in addressing health inequalities.

Study participants believed that having capacity to provide longer consultations was central to improving healthcare delivery to patients with complex needs and felt additional resourcing allowed them to focus more on coordinated and preventive healthcare practices. This is consistent with recent literature that suggests longer consultation times are associated with higher levels of both GP and patient satisfaction.^21,22^ Mercer *et al* evaluated the CARE Plus intervention, which included multiple supports for practices in very disadvantaged areas, and identified that resourcing longer consultations for complex patients in these areas was associated with an increase in patient enablement and GPs felt better equipped to provide anticipatory and coordinated care.^21^ The CARE Plus intervention was found to be cost-effective within the UK thresholds for funding of healthcare interventions. Patients themselves have identified a preference for longer consultations also.^23^

### Implications for practice

The application process for the social deprivation grant was viewed to be labour intensive and possibly prohibitive. Concerns were raised about the difficulty in planning expenditure and maintaining services because of lack of continuity of funding and delays in payment. This could be addressed through central identification of eligible practices, using routine patient and practice data and this is currently being planned within the HSE.

Evidence from the UK suggests that simply weighting per-capita GP payments for deprivation is not sufficient to address health inequity.^9,16–18^ Weighted capitation is a fundamental step towards addressing the inverse care law, but our study indicates that the addition of tailored grant supports for practices in urban deprived areas can improve patient care and sustain GPs in challenging areas of practice.

Qualitative data identified staff recruitment as an important issue for participating practices. While general practice recruitment is recognised to be an issue nationally,^24–27^ study participants identified that it is particularly challenging in areas of socioeconomic deprivation. The RoI has 29% fewer GPs per head of population than the UK and the HSE predicts a shortage of between 493–1380 GPs by the year 2025.^27^ The Irish College of General Practitioners has identified the need for workforce planning and expansion in their strategy document *Shaping the Future*.^24^ The North Dublin City GP Training programme was established in 2009 to specifically train GPs to work in areas of social deprivation and has had significant success in recruiting GPs to work in areas of deprivation.^28^ Difficulties in recruiting staff in areas of deprivation could potentially limit the ability of practices to attract the necessary staff to provide their chosen resource supported by additional funding.

In conclusion, implementation of additional resourcing for general practice in areas of socioeconomic deprivation may enhance healthcare delivery in these areas, improve staff satisfaction, and reduce the risk of burnout.

## Data

The dataset relied on in this article is available from the corresponding author on reasonable request.
